# Supplementary material for: Comprehensive Annotation and Functional Exploration of MicroRNAs in Lettuce
Source: Front Plant Sci. 2021 Dec 24;12:781836. doi: 10.3389/fpls.2021.781836 (PMC8739914; doi:10.3389/fpls.2021.781836)
Supplement: Supplementary file 3 [file Data_Sheet_9.ZIP › GSM2230752.plot/Lsa-miRN48_Lsat_1_v5_gn_9_41900.1_1645_TPlot.pdf]

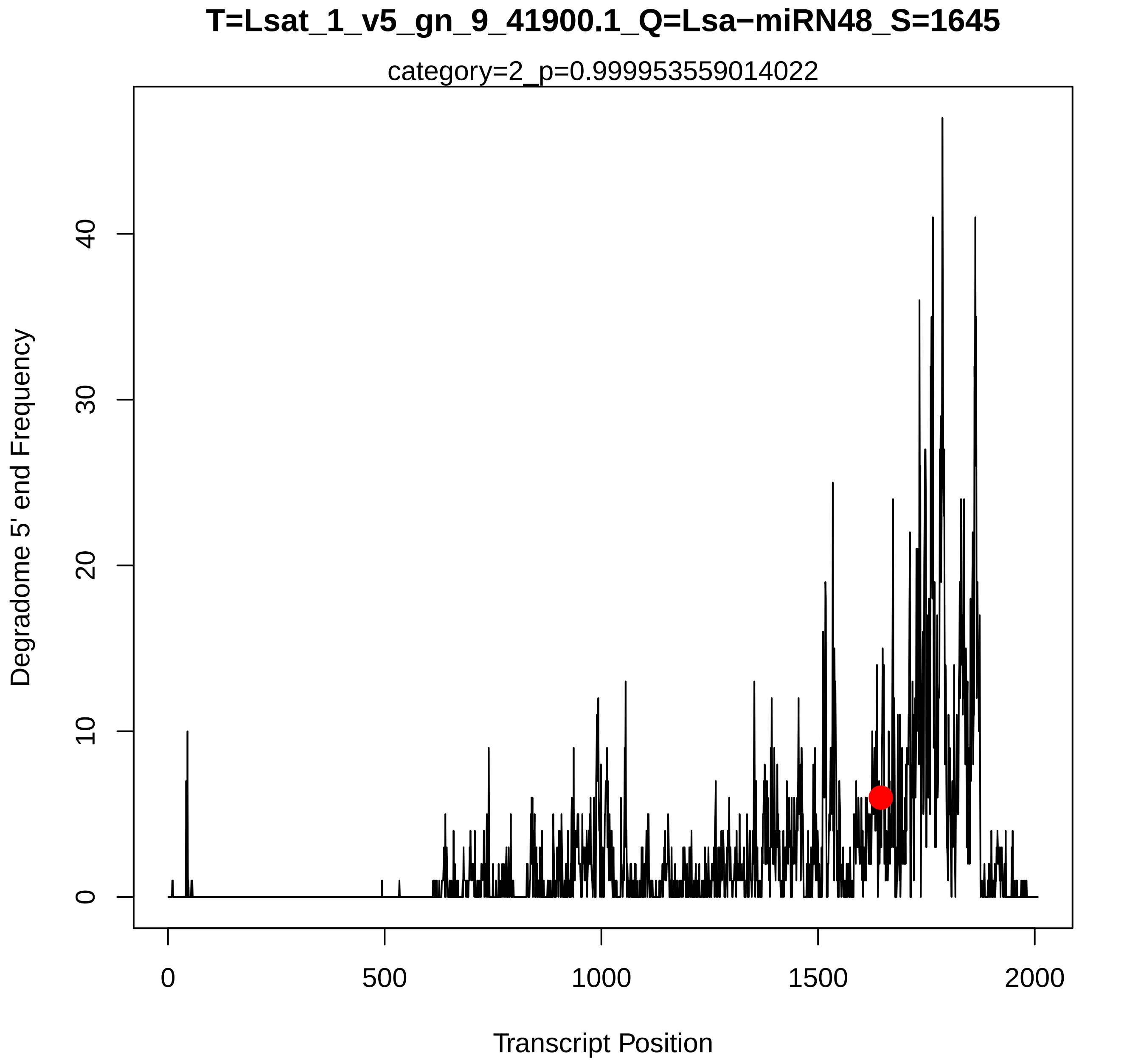

T=Lsat_1_v5_gn_9_41900.1_Q=Lsa−miRN48_S=1645
category=2_p=0.999953559014022
Degradome 5' end Frequency
0
10
20
30
40
0
500
1000
1500
2000
Transcript Position
